# Infective Aortic Valve Endocarditis Causing Embolic Consecutive ST-Elevation Myocardial Infarctions

**DOI:** 10.1155/2019/2487616

**Published:** 2019-10-14

**Authors:** Kanksha Peddi, Alexander L. Hsu, Tomas H. Ayala

**Affiliations:** ^1^Saba University School of Medicine, Saba, Dutch Caribbean, Netherlands; ^2^Department of Medicine, MedStar Harbor Hospital, Baltimore, MD, USA

## Abstract

ST-elevation myocardial infarction (STEMI) is a rare and potentially fatal complication of infective endocarditis. We report the ninth case of embolic native aortic valve infective endocarditis causing STEMI and the first case to describe consecutive embolisms leading to infarctions of separate coronary territories. Through examination of this case in the context of the previous eight similar documented cases in the past, we find that infective endocarditis of the aortic valve can and frequently affect more than a single myocardial territory and can occur consecutively. Further, current treatment modalities for embolic infective endocarditis causing acute myocardial infarction are limited and unproven. This index case illustrates the potential severity of complications and the challenges in developing standardized management for such patients.

## 1. Introduction

Infective endocarditis as a result of injection drug use is an increasingly prevalent condition in the United States [1]. With the increase in frequency of injection use-associated endocarditis, there is an associated increase in the complications associated with the disease. Symptomatic embolisms are a well-known complication of infective endocarditis (IE). Prior studies have reported embolisms in 13-49% of IE patients [2]. However, embolism to the coronary arteries is much rarer, and of these cases, even fewer describe ST-elevation myocardial infarctions (STEMIs) caused by such emboli [2]. We report a case of a unique nature: two consecutive STEMIs caused by infective endocarditis of the aortic valve. A thorough review of current literature yields only eight other previously reported cases of STEMI caused by embolic infective endocarditis of a native aortic valve [3–10]. Here, we discuss the ninth case of embolic native aortic valve infective endocarditis causing STEMI and the first case to describe consecutive embolisms leading to infarctions of separate coronary territories.

## 2. History of Presentation

A 31-year-old female with a past medical history of intravenous drug abuse (IVDA) presented with a four-day history of generalized weakness, shortness of breath, and chest pain. She described her chest pain as pleuritic and associated with a nonproductive cough. She also reported fevers with diffuse body and joint pains. The patient injected heroin daily but denied any other substance abuse. On arrival to the emergency department, the patient was afebrile, tachycardic, normotensive, and saturating well on room air. Physical exam was significant for a chronic appearing left lateral forearm wound that was approximately five by five centimeters without drainage or significant erythema. Another chronic appearing wound was found on the left lateral calf that was approximately five by seven centimeters with several foci of purulent drainage with edema and erythema of the surrounding area. Imaging later revealed a five by twelve by ten millimeter abscess in the left lateral calf. She had multiple stigmata of injection drug use along her bilateral upper and lower extremities. On this encounter, the patient was admitted for treatment of sepsis secondary to cellulitis but elected to leave against medical advice from the emergency department and was provided with a course of doxycycline and sulfamethoxazole-trimethoprim. One day following the encounter, blood cultures drawn at the time of the encounter grew methicillin-resistant Staphylococcus aureus (MRSA).

Three days following the initial encounter, the patient again presented to the emergency department. Upon arrival, the patient was confused with nonsensical speech and was unable to provide medical history. She was febrile at 39.4°C. Physical examination was notable for pale conjunctiva, jugular venous distension, tachypnea, tachycardia, a one out of six systolic ejection murmurs at the right upper sternal border, and extensive skin wounds as described prior.

### 2.1. Past Medical History

The patient is with a past medical history of intravenous drug use.

### 2.2. Investigations

Initial blood work of the second encounter was significant for a white blood cell count of 33.1 k/*μ*L, hemoglobin of 6.2 g/dL, hematocrit of 18.4%, mean corpuscular volume of 69.2 fL, absolute neutrophil count of 29.3 k/*μ*L, sodium of 132 mmol/L, potassium of 3.4 mmol/L, bicarbonate of 18 mmol/L, blood urea nitrogen of 27 mg/dL, troponin of 30.5 ng/mL, N-terminal pro-B-type natriuretic peptide of 27,028 pg/mL, cocaine-positive urine toxicology, and lactic acid of 3.1 mmol/L.

### 2.3. Differential Diagnosis

- Infective endocarditis
- Cellulitis, abscess
- Opioid overdose
- Drug-induced vasospasm

### 2.4. Imaging

**Figure 1:**
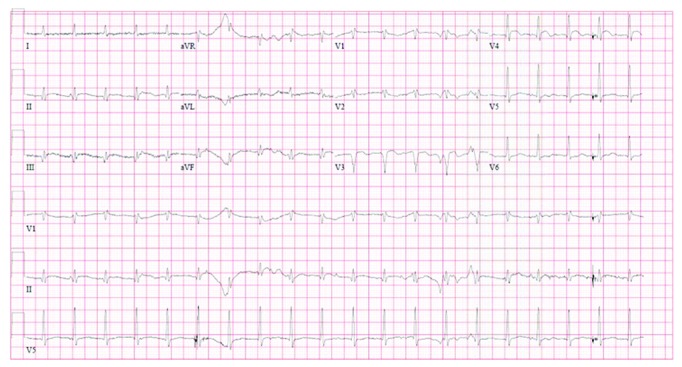
Electrocardiogram performed on patient arrival demonstrating ST-elevation in leads V3-V4.

**Figure 2:**
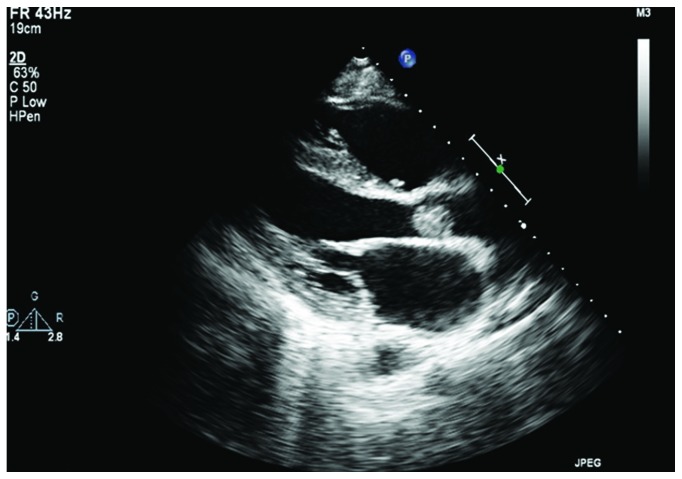
Echocardiogram demonstrating a vegetation measuring 1.8 cm × 1.3 cm.

**Figure 3:**
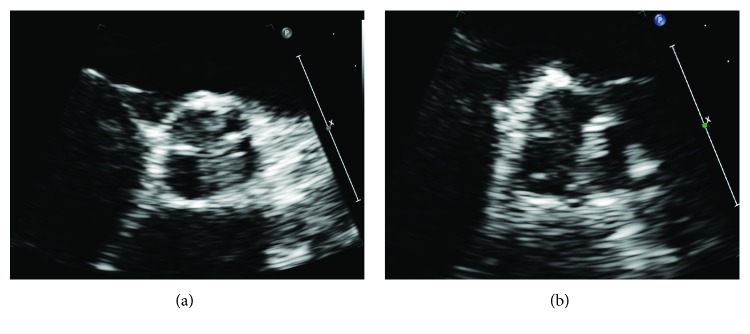
Echocardiogram demonstrating a vegetation proximal to the left aortic cusp (a) and the vegetation mobilizing toward the left coronary artery origination anastomosis upon valve opening (b).

An electrocardiogram performed on arrival demonstrated an anterolateral STEMI (Figure 1). Echocardiogram revealed anterior wall motion abnormalities and an aortic valve vegetation that filled more than half of the left ventricular outflow tract measuring 1.8 by 1.3 centimeters (Figures 2 and 3).

### 2.5. Management

**Figure 4:**
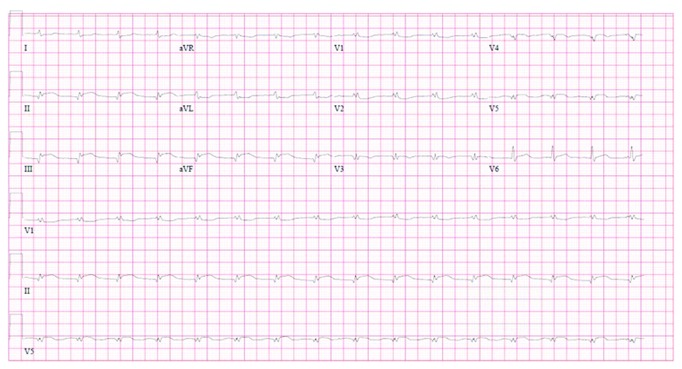
Electrocardiogram performed five hours after first electrocardiogram (Figure 1) demonstrating ST-elevation in leads II, III, and AVF.

The patient was started on vancomycin, piperacillin, tazobactam, and ceftriaxone. Acute cardiac intervention, which would have required transfer to a tertiary care center with cardiothoracic surgery capabilities, was deferred due to the patient's unstable hemodynamic state and comorbid conditions. A repeat electrocardiogram five hours later as the patient became more hypotensive revealed evolution of anterior MI with diffuse Q waves and an inferior lead STEMI (Figure 4). In the following hours, the patient became more hypotensive and hypoxic and required vasopressors and intubation with ventilator support. Subsequently, the patient became pulseless requiring cardiopulmonary resuscitation with return of spontaneous circulation after six minutes of advanced cardiac life support. Approximately twelve hours after presentation, the patient expired from cardiovascular collapse. An autopsy was not performed postmortem.

## 3. Discussion

There are few documented cases of acute myocardial infarction caused by IE particularly of the aortic valve. In IVDA patients, infective endocarditis most frequently affects tricuspid valves (50%) and aortic (20%) and mitral valves (20%) less frequently [11]. Most emboli stem from mitral valve vegetations and in patients with prior valve replacement. This case is unique in that the patient presented with a native aortic valve vegetation that embolized to cause two consecutive acute ST-elevation myocardial infarctions affecting two distinct coronary artery territories. However, it is also necessary to consider two factors that increase the likelihood of embolization unique to this case: vegetation length and microbiology. In 2018, Mahananey et al. suggest that vegetation size of greater than 10 mm may be associated with increased embolization risk [12]. In the case presented, the patient had an 18 mm vegetation [12]. Further, Hubert et al. find that Staphylococcus aureus is also associated with an increased risk of embolization, which conforms to the microbiology found in this case [13].

There are eight previously documented cases of ST-elevation myocardial infarctions caused by embolic native aortic valve endocarditis [3–10]. The cases illustrate a spectrum of embolized coronary vessels and corresponding myocardial territories. Including the case presented here, infarcted myocardial territories are nearly all equally represented with several cases describing multiple affected territories [3–10]. In the documented cases, 3/9 demonstrate anterior myocardial infarctions [4, 5], 2/9 demonstrate anterolateral infarctions [7, 10], 4/9 demonstrate inferior territory infarctions [6, 8, 10], and 1/9 demonstrates lateral territory infarction [9]. While there are other cases that demonstrate multiterritory infarcted myocardium, we present the first case to illustrate two consecutive infarctions. Further, infective endocarditis of the aortic valve can and frequently affect more than a single myocardial territory and can occur consecutively. This case serves as notice for clinicians to remain vigilant of the potential for sequential STEMIs in these patients.

Current treatment modalities for embolic infective endocarditis causing acute myocardial infarction are limited and unproven. Detailed guidelines for managing STEMI and for managing infective endocarditis as separate conditions exist but recommendations for concurrent diagnoses are lacking. The treatment modalities applied in the nine cases of STEMI caused by embolic aortic valve endocarditis varied considerably, as did the affected patient characteristics and associated outcomes. Of the nine cases, three underwent angioplasty; two of the three were reported to survive beyond the immediately observed period [4, 5, 7]. Three patients underwent aortic valve replacement with two surviving beyond the observed period; in one case, the final survival status was not reported [6, 9, 10]. One patient was administered fibrinolytic therapy and survived with complication of a major gastrointestinal bleed [3]. Finally, in two cases, including the one presented here, the patient received neither invasive nor fibrinolytic therapy; one patient reportedly survived beyond the observed period [8]. Given the small treatment group sizes and the infrequency of the condition, it is difficult to draw conclusions regarding treatment superiority. It is essential to evaluate each patient individually, particularly as there are no clear guidelines to direct clinicians in managing patients presenting with myocardial infarction in the setting of infective endocarditis.

Due to the rarity of this case, we consider possible predisposing factors as well as an alternative explanation to this presentation. Rather than two distinct emboli affecting different vessels, it is necessary to consider the possibility that a single embolus propagated through a wrap-around left anterior descending (LAD) artery supplying both the anterior and inferior cardiac territories. Indeed, such a scenario could feasibly present as consecutive STEMI of different coronary myocardial territories. However, while a definitive answer attained through perhaps a coronary angiogram is not available in this case, we believe the presented electrocardiograms and echocardiogram support that there were indeed two distinct emboli. First, in the initial electrocardiogram (Figure 1), the absence of inferior ischemic findings with an upstream blockage lends support that the inferior myocardial territory is not supplied by a wrap-around LAD but likely rather supplied by the right coronary artery (RCA). This is further reinforced by an echocardiogram acquired during the first insult that revealed only anterior wall motion abnormality without evidence of apical or inferior wall motion abnormality. Finally, there would likely be preferential embolization to the RCA following injury to the flow of the anterior territory.

## 4. Conclusion

Among the reported cases of infective aortic valve endocarditis causing embolic acute myocardial infarction, we present the first case with consecutive infarctions involving separate myocardial territories. This index case illustrates the potential severity of complications and the challenges in developing standardized management for such patients. Through this case, we hope to expand the fund of knowledge and judicious approach for similar future patients.
